# Epidemic 2014 Enterovirus D68 Cross-Reacts with Human Rhinovirus on a Respiratory Molecular Diagnostic Platform

**DOI:** 10.1371/journal.pone.0118529

**Published:** 2015-03-23

**Authors:** Shane C. McAllister, Mark R. Schleiss, Sophie Arbefeville, Marie E. Steiner, Ryan S. Hanson, Catherine Pollock, Patricia Ferrieri

**Affiliations:** 1 Division of Pediatric Infectious Diseases and Immunology, University of Minnesota Medical School, Minneapolis, Minnesota, United States of America; 2 Center for Infectious Disease and Microbiology Translational Research, University of Minnesota Medical School, Minneapolis, Minnesota, United States of America; 3 Department of Laboratory Medicine and Pathology, University of Minnesota Medical School, Minneapolis, Minnesota, United States of America; 4 Division of Pediatric Hematology and Oncology, University of Minnesota Medical School, Minneapolis, Minnesota, United States of America; 5 Division of Pediatric Critical Care, University of Minnesota Medical School, Minneapolis, Minnesota, United States of America; Kliniken der Stadt Köln gGmbH, GERMANY

## Abstract

Enterovirus D68 (EV-D68) is an emerging virus known to cause sporadic disease and occasional epidemics of severe lower respiratory tract infection. However, the true prevalence of infection with EV-D68 is unknown, due in part to the lack of a rapid and specific nucleic acid amplification test as well as the infrequency with which respiratory samples are analyzed by enterovirus surveillance programs. During the 2014 EV-D68 epidemic in the United States, we noted an increased frequency of “low-positive” results for human rhinovirus (HRV) detected in respiratory tract samples using the GenMark Diagnostics eSensor respiratory viral panel, a multiplex PCR assay able to detect 14 known respiratory viruses but not enteroviruses. We simultaneously noted markedly increased admissions to our Pediatric Intensive Care Unit for severe lower respiratory tract infections in patients both with and without a history of reactive airway disease. Accordingly, we hypothesized that these “low-positive” RVP results were due to EV-D68 rather than rhinovirus infection. Sequencing of the picornavirus 5’ untranslated region (5’-UTR) of 49 samples positive for HRV by the GenMark RVP revealed that 33 (67.3%) were in fact EV-D68. Notably, the mean intensity of the HRV RVP result was significantly lower in the sequence-identified EV-D68 samples (20.3 nA) compared to HRV (129.7 nA). Using a cut-off of 40 nA for the differentiation of EV-D68 from HRV resulted in 94% sensitivity and 88% specificity. The robust diagnostic characteristics of our data suggest that the cross-reactivity of EV-D68 and HRV on the GenMark Diagnostics eSensor RVP platform may be an important factor to consider in making accurate molecular diagnosis of EV-D68 at institutions utilizing this system or other molecular respiratory platforms that may also cross-react.

## Introduction

Enterovirus D68 (EV-D68) was first isolated in 1962 and is known to cause sporadic disease and limited outbreaks of severe lower respiratory tract infections, predominantly in children. In August 2014 the Centers for Disease Control received notification of clusters of severe respiratory disease in children and increased identification of enterovirus/rhinovirus positive samples on multiplex nucleic acid amplification assays [1]. Sequencing by the CDC Picornavirus Laboratory identified EV-D68 in samples submitted by hospitals in Missouri and Illinois [1]. In the following weeks, EV-D68 was identified in other Midwestern and Northeastern states and as of October 29, 2014, the CDC had reported EV-D68 in 47 states. However, the lack of a readily available, rapid diagnostic test for EV-D68 makes it difficult to ascertain the true extent of the current epidemic.

At the University of Minnesota Children’s Hospital in Minneapolis, Minnesota, we noted an abrupt increase in children admitted to our Pediatric Intensive Care Unit (PICU) with severe asthma exacerbations in late August. As in other descriptions of the clinical course of infection with the 2014 epidemic strain(s) of EV-D68, we also noted a sudden increase in admissions to the PICU of children with no documented prior history of wheezing. Many children admitted to the PICU with symptoms of severe respiratory infection had nasopharyngeal swab specimens analyzed in our clinical microbiology laboratory using the GenMark Diagnostics eSensor respiratory virus panel (RVP) platform. Of the 14 viruses detected by this multiplex nucleic acid amplification assay we noted numerous “low-positive” results for human rhinovirus (HRV), i.e., less than 20 nanoamperes (nA), whereas positive control reference strains on this platform are routinely greater than 150 nA. This prompted us to analyze these “low-positive” samples in further detail and compare them with “high-positive” HRV results.

Of template purified from 50 samples that were positive for HRV on the GenMark Diagnostics eSensor, 49 yielded PCR products using primers designed for sequencing of EV-D68; sequencing of these PCR products demonstrated that 33 of these were EV-D68 rather than HRV. The significance of this is that outbreaks of closely-related picornavirus infections might yield “false positive” results with nucleic acid amplification tests, and as a result, the importance of integrating epidemiological data with clinical observations and molecular diagnostics must be emphasized. Furthermore, given the likely under-reporting of respiratory infections caused by enterovirus, inclusion of targets specific for enteroviruses in future iterations of respiratory molecular diagnostic panels should be considered.

## Results

### PCR amplification of the 5’-UTR of EV-D68

**Fig 1:**
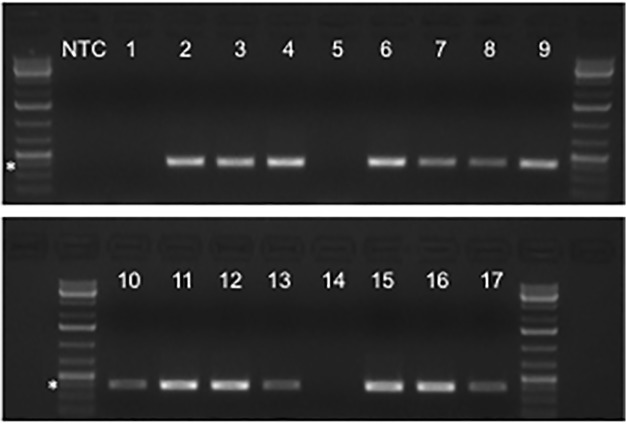
PCR amplification of a portion of the 5’-UTR from GenMark eSensor RVP samples. cDNA synthesized from RNA extracted from de-identified and masked RVP samples was used for traditional PCR amplification of the 5’-UTR. Primers were described by Oberste, *et*. *al*., for the sequencing of EV-D68 [2]. Agarose-ethidium bromide electrophoresis demonstrated that 49 of 62 samples analyzed yielded a 396 base pair amplicon compatible with EV-D68. Results shown corresponded to samples 2–4, 6–13, and 15–17, and are representative of the range of band intensities observed for all positive samples. NTC: no template control.

All samples available for analysis were assayed by traditional PCR in order to detect the picornavirus 5’-UTR using primers described by Oberste, *et*. *al*., (Fig. 1) [2]. Of 62 de-identified/masked RVP samples analyzed, 49 yielded the expected 396 base pair amplicon (Table 1). Upon un-masking we found that all PCR-positive samples had been positive for HRV on the GenMark eSensor RVP platform. By comparing the RVP results with the samples that did not yield the expected 396 base pair amplicon, we found that only one sample was positive for HRV (sample 1), one was positive for parainfluenza type 3 (sample 21), and one was positive for adenovirus (sample 38). One sample that was RVP-positive for both HRV and adenovirus (sample 3) yielded the expected amplicon, confirming that co-infection with more than one detectable virus did not interfere with the performance of the GenMark eSensor RVP platform or our PCR analysis. During the period of study none of the samples submitted to the UMN clinical microbiology laboratory was positive on the RVP platform for influenza A or B, human metapneumovirus, parainfluenza virus types 1 or 2, or respiratory syncytial virus A or B. Notably, none of the ten RVP-negative samples yielded PCR amplicons (samples 5, 14, 26, 37, 42, 46, 48, 49, 57, and 60). Together these data supported the specificity of the PCR primers for the *Picornaviridae* 5’-UTR.

**Table 1:** RVP Signal, 5'-UTR PCR, and sequencing results.

Sample no.	RVP*	PCR	Sequence	Sample no.	RVP*	PCR	Sequence
1	HRV 71.9	−	-	32	HRV 163.4	+	HRV
2	HRV 11.8	+	EV-D68	33	HRV 6.7	+	HRV
3	HRV 202.1	+	HRV	34	HRV 178.2	+	HRV
	Adeno 139.6			35	HRV 30	+	EV-D68
4	HRV 208.9	+	HRV	36	HRV 23	+	EV-D68
5	-	−	-	37	-	−	-
6	HRV 13.7	+	EV-D68	38	Adeno 68.2	-	-
7	HRV 19.9	+	EV-D68	39	HRV 5.5	+	EV-D68
8	HRV 11.8	+	EV-D68	40	HRV 11.4	+	EV-D68
9	HRV 6	+	EV-D68	41	HRV 17.8	+	EV-D68
10	HRV 5.9	+	EV-D68	42	-	−	-
11	HRV 19.8	+	EV-D68	43	HRV 15	+	EV-D68
12	HRV 22.1	+	EV-D68	44	HRV 28.4	+	EV-D68
13	HRV 21.3	+	EV-D68	45	HRV 46.7	+	EV-D68
14	-	−	-	46	-	−	-
15	HRV 164.9	+	HRV	47	HRV 3.4	+	EV-D68
16	HRV 15.1	+	EV-D68	48	-	−	-
17	HRV 6.9	+	EV-D68	49	-	−	-
18	HRV 142.6	+	HRV	50	HRV 10.4	+	EV-D68
19	HRV 28.8	+	EV-D68	51	HRV 182.8	+	HRV
20	HRV 115	+	HRV	52	HRV 37.9	+	EV-D68
21	PIV3	−	-	53	HRV 23.1	+	EV-D68
22	HRV 67.2	+	HRV	54	HRV 29.2	+	HRV
23	HRV 16.2	+	EV-D68	55	HRV 183.6	+	HRV
24	HRV 37.6	+	EV-D68	56	HRV 90.9	+	HRV
25	HRV 73	+	HRV	57	-	−	-
26	-	−	-	58	HRV 12.6	+	EV-D68
27	HRV 9.1	+	EV-D68	59	HRV 12.6	+	EV-D68
28	HRV 79.5	+	EV-D68	60	-	−	-
29	HRV 35.2	+	EV-D68	61	HRV 9.7	+	EV-D68
30	HRV 23.2	+	EV-D68	62	HRV 42.4	+	HRV
31	HRV 224.3	+	HRV				

*HRV: human rhinovirus, Adeno: adenovirus, EV-D68 enterovirus D68, PIV3: parainfluenza virus type 3. Number adjacent to RVP result indicates signal strength on GenMark eSensor RVP platform

### Sequence identity of PCR-positive samples

The 49 samples that yielded an amplicon using 5’-UTR primers were submitted for Sanger sequencing. All of these samples yielded good-quality sequences. BLAST searches of the 5’-UTR sequences demonstrated that 33 PCR-positive samples were EV-D68 and 16 were HRV (Table 1). Within the 33 EV-D68-positive samples, there were 14 unique sequences identified. Three of these sequences were shared by more than one EV-D68-positive sample, and one representative sequence for each of these groups was submitted to GenBank. The remaining 11 sequences that were identified only once were also submitted to GenBank. The most commonly identified sequence was shared by samples 2, 7, 9, 11, 12, 13, 16, 17, 27, 29, 30, 35, 39, 44, 45, 47, 52, 53 (sample 13 accession number KP055080). Samples 24 and 50 were identical (sample 24 accession number KP055081), as were samples 6 and 61 (sample 6 accession number KP055082). Unique sequences were identified for 8, 10, 19, 23, 28, 36, 40, 41, 43, 58, and 59 (accession numbers KP055083, KP055084, KP055085, KP055086, KP055087, KP055088, KP055089, KP055090, KP055091, KP055092, KP055093, respectively). Pairwise alignment of these 33 sequences revealed sequence identity ranging from 97.9 to 100%, while alignment of each isolate to the consensus sequence ranged from 98.8 to 100% (Table 2). Alignment with our consensus sequence revealed more distant relatedness to the other group D enteroviruses EV94 and EV70 (84.1% and 84%, respectively), while sequence identity to other published EV-D68 isolates ranged from 90.5 to 99.1%, with higher identity noted in more recently published sequences.

**Table 2:** Percent 5'-UTR Fragment Sequence Identity.

UMN strains compared pairwise:			97.9 to 100%
UMN strains compared to consensus sequence:			98.8 to 100%
UMN consensus sequence compared to:			
Virus	Strain	GenBank	
EV94	E210	DQ916376.1	84.1
EV70	J670/71	DQ201177.1	84
EV-D68	NYC403	JX101846.1	95.6
EV-D68	NYC399	JX101818.1	90.5
EV-D68	GA427	JX101838.1	91.1
EV-D68	NZ-2010-541	JX070222.1	95.6
EV-D68	91106975	JX310688.1	95.3
EV-D68	37–99	EF107098.1	96.4
EV-D68	JPOC10-378	AB601883.2	96.2
EV-D68	JPOC10-290	AB601882.2	96.7
EV-D68	CU171	KM361524.1	97.9
EV-D68	CU134	KM361523.1	97.6
EV-D68	US/MO/14-18950	KM851228.1	98.8
EV-D68	US/MO/14-18949	KM851227.1	99.1
EV-D68	US/MO/14-18948	KM851226.1	98.5
EV-D68	US/MO/14-18947	KM851225.1	99.1
EV-D68	US/KY/14-18953	KM851231.1	96.2
EV-D68	BCH895A	KF726085.1	95.6
EV-D68	Fermon	AY426531.1	94.4

UMN: University of Minnesota

**Fig 2:**
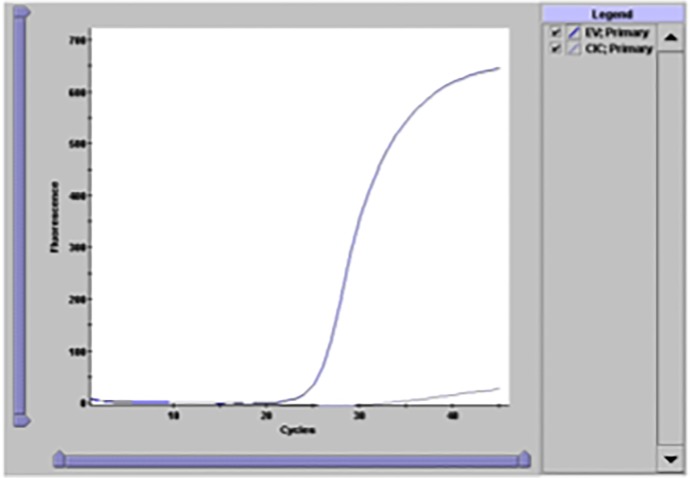
Detection of enterovirus in sample 13 using the GeneXpert enterovirus assay. As independent confirmation of the sequencing results for sample 13, we analyzed extracted nucleic acid on the GeneXpert platform. Sample 13, which had a RVP signal for HRV of 21.3 nA, gave a positive signal (blue line) with a C_T_ of 24.2 and an excellent amplification curve consistent with positive results obtained from CSF samples, whereas the internal control (CIC) amplified less efficiently, as expected, because of competition (gray line).

As an independent confirmation that we were correctly identifying enterovirus in our sample set, we analyzed sample 13, which was identified as EV-D68 by our sequencing, using the GeneXpert enterovirus assay employed in our clinical microbiology laboratory for detection of enterovirus nucleic acid in cerebrospinal fluid (CSF) samples. Though this assay was not validated for detection of enterovirus from other sample types, we were able to detect enterovirus in sample 13 with a similar amplification curve as expected for positive CSF samples (Fig. 2).

### RVP signal strength for sequence-confirmed EV-D68 compared with HRV isolates

**Fig 3:**
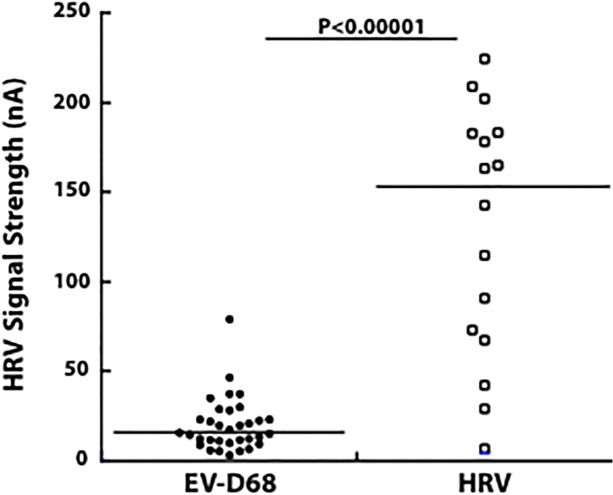
GenMark eSensor RVP signal strength for HRV in samples stratified by sequence results. Un-masking of the RVP results for all 62 samples revealed that the 49 positive PCR amplicons identified were derived from those samples that were also positive for HRV on the GenMark panel. Sequencing of these amplicons revealed that 33 were EV-D68 and 16 were HRV. The mean signal strength in nA of the RVP results for those samples identified as EV-D68 was significantly lower than for the HRV samples (20.3 nA versus 129.7 nA, respectively, p < 0.00001).

To test our hypothesis that samples with “low-positive HRV” results on the GenMark eSensor RVP assay were in fact EV-D68, we compared the mean signal intensity of RVP results between the sequence-confirmed EV-D68 and HRV samples (Fig. 3). The mean RVP signal intensity of 33 EV-D68 isolates was 20.3 nA (range 3.4 to 79.5, median 16.2) compared to 129.7 nA of 16 HRV isolates (range 6.7 to 224.3, median 153) (p < 0.00001).

Finally, we evaluated the performance characteristics of the RVP platform for differentiating EV-D68 from HRV in clinical samples. Using cut-offs of 40 nA (Table 3) or 50 nA (Table 4) to define EV-D68 in samples yielding positive results for HRV on the RVP platform, we calculated sensitivity, specificity, as well as positive- and negative-predictive values. Based on these data, we concluded that a cut-off of 40 nA defined the best parameter for distinguishing EV-D68 from HRV using the GenMark platform.

**Table 3:** Differentiating EV-D68 from HRV by RVP signal of 40Na.

	EV68 Sequenced				HRV Sequenced		
	Yes	No			Yes	No	
RVP ≤50	32	3	35	RVP ≥50	13	2	15
RVP>50	1	14	15	RVP<50	3	32	35
	33	17			16	34	
Sensitivity	97%			Sensitivity	81%		
Specificity	82%			Specificity	94%		
PPV	91%			PPV	87%		
NPV	93%			NPV	91%		

**Table 4:** Differentiating EV-D68 from HRV by RVP signal of 50nA.

	EV68 Sequenced				HRV Sequenced		
	Yes	No			Yes	No	
RVP ≤40	31	2	33	RVP ≥40	14	3	17
RVP>40	2	15	17	RVP<40	2	31	33
	33	17			16	34	
Sensitivity	94%			Sensitivity	88%		
Specificity	88%			Specificity	91%		
PPV	94%			PPV	82%		
NPV	88%			NPV	94%		

## Discussion

EV-D68 was first isolated in 1962 and was categorized as a group D human enterovirus based on sequencing [3]. Biochemically, EV-D68 shares some common features with HRVs including acid sensitivity and growth at lower optimal temperature than for other enteroviruses [4]. Blomqvist, *et*. *al*., also demonstrated that EV-D68 could be neutralized by monotypic antiserum specific for HRV87 confirming that these two viruses represent strains of the same picornavirus [4]. The shared biological characteristics of EV-D68 and rhinoviruses and their cross-reactivity in many nucleic acid amplification tests make the true prevalence of EV-D68 infection difficult to determine.

Following initial identification in 1962, EV-D68 was isolated only sporadically in the United States until 2005 [5]. An ascertainment bias may in part explain the rarity of EV-D68 identification since CSF and stool samples are far more likely than respiratory samples to be assayed for enteroviruses [5]. Data reported by Smura, *et*. *al*., suggests that EV-D68 may be far more common than surveillance studies suggest. Of the 281 serum samples collected from the Finnish Maternity Cohort, 100% were positive for EV-D68-specific antibodies, whereas only 79.4% and 16.7% were positive for other group D enteroviruses EV94 and EV70, respectively [6].

Meijer, *et*. *al*., analyzed data from 1994 through 2010 collected by a national respiratory infection surveillance program and reported a total of 71 samples positive for EV-D68 out of 13,310 samples collected (0.5%) [7]. Notably, 24 of the 71 EV-D68-positive samples were from 2010 and were found to have higher sequence diversity in the VP1 gene compared with previous isolates, confirming that the increased detection of EV-D68 in 2010 was due to an epidemic. Similar studies have reported contemporaneous outbreaks in North America, Europe, Asia, and Africa suggesting that EV-D68 is an emerging pathogen [8–16]. Improved molecular diagnostics capable of identifying EV-D68 will help clarify the true extent of EV-D68 infection and disease.

Cross-reactivity of different nucleic acid amplification assays for enteroviruses and rhinoviruses has been reported previously [16–18]. The GenMark platform amplifies a portion of the 5’-UTR region of HRV. Within viral particles the 5’-UTR is covalently bound to the VPG protein, which secures the viral genome to the viral capsid. Importantly, the 5’-UTR also contains the internal ribosomal entry site (IRES), which mediates translation from the un-capped, positive-sense RNA genome [19]. Mutations in this region have been described in association with outbreaks, suggesting that this region may influence virulence [20]. Phylogenetic analysis of picornaviruses based on the 5’-UTR demonstrates high sequence identity among EV-D68 isolates and to a lesser degree between EV-D68 and other group D enteroviruses [2]. HRV isolates are even more distantly related based on the 5’-UTR, though primers used to amplify the picornavirus 5’-UTR differ between diagnostic platforms and the choice of amplified region can effect cross-reactivity. Here we have demonstrated cross-reactivity of EV-D68 and HRV on the GenMark platform; however, further work will be necessary to ascertain cross-reactivity with other respiratory enteroviruses such as EV70 and EV94.

This 2014 epidemic EV-D68 outbreak illustrated the challenges in a diagnostic microbiology/virology laboratory of identifying with certainty a viral etiology for patients with serious respiratory illnesses with wheezing. Since it was unlikely, in our opinion, that low signal HRV results from the GenMark eSensor instrument indicated an epidemic of rhinovirus disease, we pursued the hypothesis that these might be cross-reacting signals for a related picornavirus. Using Sanger sequencing, we were able to confirm that these samples contained EV-D68, which we were unable to isolate in cell culture. By combining epidemiological data with molecular technology, we unraveled the explanation for the initial respiratory virus PCR results. It is possible that the current 2014 EV-D68 strain differs genetically in a key region from previous EV-D68 strains, and that this difference may account for heightened virulence in children.

## Methods

### Evaluation of patient samples in clinical microbiology laboratory

The GenMark eSensor Respiratory Viral Panel (RVP) (GenMark Dx, Carlsbad, CA) is an FDA-approved multiplexed nucleic acid test that uses a solid-phase electrochemical method on the eSensor XT-8 platform for detection of nucleic acid targets. Targets are detected by voltage change when DNA binds to capture probes on a gold electrode. This panel includes primers and signal probes for adenovirus species B/E (combined result), and species C; influenza virus A, including subtypes H1, H3, and 2009 H1N1; influenza B virus; human metapneumovirus; parainfluenza virus types 1, 2, and 3; respiratory syncytial virus subtypes A and B; and human rhinovirus. Specimens were tested according to the manufacturer’s instructions. The sample nucleic acids were extracted on the NucliSENS easyMAG instrument (bioMérieux, Boxtel, The Netherlands). The conventional endpoint PCR and the exonuclease steps were performed on an Applied Biosystems 2720 Thermal Cycler (Applied Biosystems, Foster City, CA). The detection, data acquisition, and automated analysis steps were performed on the eSensor XT-8 instrument. The human rhinovirus assay primers and probe(s) amplify and detect a portion of the 5’-untranslated region (5’-UTR) (http://www.accessdata.fda.gov/cdrh_docs/pdf11/K113731.pdf). Results are expressed as nanoamperes (nA).

The GeneXpert enterovirus assay (Cepheid, Sunnyvale, CA) is an FDA-approved assay designed to detect enterovirus (EV) RNA in cerebrospinal fluid samples. The GeneXpert Dx System automates and integrates sample purification, nucleic acid amplification, and detection of the target sequence using real-time PCR and RT-PCR assays. The system employs single-use disposable GeneXpert cartridges that hold the PCR reagents and host the PCR process. The EV primers and probe amplify and detect a consensus region of the enterovirus genome 5’-UTR between nucleotide 452 and 596 (http://www.accessdata.fda.gov/cdrh_docs/pdf6/K061062.pdf and http://www.accessdata.fda.gov/cdrh_docs/reviews/K061062.pdf). The sample nucleic acids were extracted on the NucliSENS easyMAG instrument using the same protocol as for the GenMark eSensor Respiratory Viral Panel assay. The extracted nucleic acid was then diluted and 140 μl of the dilution was processed in the GeneXpert cartridges. Results are expressed automatically as cycle threshold (C_T_) and amplification curves can be viewed on the linked computer.

### Detection of enterovirus D68 in clinical samples

Extracted nucleic acid from 62 consecutive nasopharyngeal swab samples submitted to the clinical microbiology laboratory at the University of Minnesota Medical School between September 4 and September 16 (during the peak epidemic of EV-D68 in Minnesota) were de-identified and the GenMark eSensor RVP results masked. Total RNA was isolated using the RNeasy Mini Prep Kit (QIAGEN, Valencia, CA) with RNase-free DNase (QIAGEN) according to the manufacturer’s protocol. RNA yields were 10 to 50 ng in 50 μl of eluent; 8 μl of purified RNA was used to synthesize cDNA using SuperScript VILO Master Mix (Invitrogen, Carlsbad, CA) in a 10 μl reaction volume according to manufacturer’s protocol.

Amplification of the 5’-UTR of was performed with HotStarTaq MM Kit (QIAGEN) in an 80 μl reaction volume using 8 μL of cDNA and a final primer concentration of 0.2 μM (forward primer 5’- CTCGGATCCCAAGCAACTTCTGTTTCCCCGG-3’ and reverse primer 5’- ACACGGACACCCAAAGTAGTCGGTTCC-3’ from Oberste, *et*. *al*., which were developed for EV-D68 sequencing [2]). Samples were cycled as follows: 95°C for 15 minutes; 94°C for 30 seconds, 60°C for 35 seconds, and 72°C for 60 seconds for 40 cycles; and a final extension at 72°C for 5 minutes. All PCR reactions were resolved on 1.5% agarose-ethidium bromide gels. For all samples that yielded the expected 396 base pair product, 50 μl of the corresponding PCR reaction were purified using the PureLink PCR purification Kit (Invitrogen) according to the manufacturer’s protocol. DNA yields were 200 to 2250 ng in 50 μl of eluent.

Samples were prepared for Sanger sequencing using the reverse primer in accordance with the University of Minnesota Genomics Center specifications (20 ng of PCR amplicon, 6.4 pmol primer, 12 μl final reaction volume), and the resulting sequence data was analyzed with Sequence Scanner Software (Applied Biosystems, Grand Island, NY). Alignment by clustalW analysis was performed with MacVector software (Cary, NC). BLAST searches of GenBank sequences were performed to identify which picornavirus was present in each PCR-positive sample.

### Viral culture

A subset of nasopharyngeal swab samples were collected and placed in viral transport medium for further analysis. These samples were inoculated on primary rhesus monkey kidney cells, the human lung carcinoma cell line A549, and a human foreskin fibroblast cell line (CellProLabs, Golden Valley, MN). Cultures were incubated at 35°C for 14 days and monitored daily for cytopathic effect.

### Statistical analysis

To determine the statistical significance of the difference in mean RVP signal strength between EV-D68 and HRV isolates a 1-way ANOVA was performed using STATA software (College Station, TX).

### Ethics statement

The samples analyzed in this report were derived from nasopharyngeal swab specimens collected from patients admitted to the PICU at the University of Minnesota Masonic Children’s Hospital. These samples were collected for analysis on the GenMark eSensor RVP platform as part of routine clinical care. Nucleic acid samples that remained after testing in our clinical microbiology laboratory were de-identified as required by our institutional review board for further analysis.

## References

[1] Midgley CM, Jackson MA, Selvarangan R, Turabelidze G, Obringer E, Johnson D, et al Severe respiratory illness associated with enterovirus d68—missouri and illinois. MMWR Morb Mortal Wkly Rep. 2014; 63: 798–799. 25211545 PMC4584696

[2] Oberste MS, Maher K, Schnurr D, Flemister MR, Lovchik JC, Peters H, et al Enterovirus 68 is associated with respiratory illness and shares biological features with both the enteroviruses and the rhinoviruses. J Gen Virol. 2004; 85: 2577–2584. 15302951 10.1099/vir.0.79925-0

[3] Schieble JH, Fox VL, Lennette EH. A probable new human picornavirus associated with respiratory diseases. Am J Epidemiol. 1967; 85: 297–310. 4960233 10.1093/oxfordjournals.aje.a120693

[4] Blomqvist S, Savolainen C, Råman L, Roivainen M, Hovi T. Human rhinovirus 87 and enterovirus 68 represent a unique serotype with rhinovirus and enterovirus features. J Clin Microbiol. 2002; 40: 4218–4223. 12409401 10.1128/JCM.40.11.4218-4223.2002 PMC139630

[5] Khetsuriani N, Lamonte-Fowlkes A, Oberst S, Pallansch MA, Centers for Disease Control and Prevention. Enterovirus surveillance—United States, 1970–2005. MMWR Surveill Summ. 2006; 55: 1–20. 16971890

[6] Smura T, Ylipaasto P, Klemola P, Kaijalainen S, Kyllönen L, Sordi V, et al Cellular tropism of human enterovirus D species serotypes EV-94, EV-70, and EV-68 in vitro: implications for pathogenesis. J Med Virol. 2010; 82: 1940–1949. 10.1002/jmv.21894 20872722

[7] Meijer A, van der Sanden S, Snijders BEP, Jaramillo-Gutierrez G, Bont L, van der Ent K, et al Emergence and epidemic occurrence of enterovirus 68 respiratory infections in The Netherlands in 2010. Virology. 2012; 423: 49–57. 10.1016/j.virol.2011.11.021 22177700

[8] Ikeda T, Mizuta K, Abiko C, Aoki Y, Itagaki T, Katsushima F, et al Acute respiratory infections due to enterovirus 68 in Yamagata, Japan between 2005 and 2010. Microbiol Immunol. 2012; 56: 139–143. 10.1111/j.1348-0421.2012.00411.x 22309616

[9] Rahamat-Langendoen J, Riezebos-Brilman A, Borger R, van der Heide R, Brandenburg A, Scholvinck E, et al Upsurge of human enterovirus 68 infections in patients with severe respiratory tract infections. J Clin Virol. 2011; 52: 103–106. 10.1016/j.jcv.2011.06.019 21802981

[10] Lauinger IL, Bible JM, Halligan EP, Aarons EJ, MacMahon E, Tong CYW. Lineages, sub-lineages and variants of enterovirus 68 in recent outbreaks. PLoS ONE. 2012; 7: e36005 10.1371/journal.pone.0036005 22536453 PMC3335014

[11] Imamura T, Suzuki A, Lupisan S, Kamigaki T, Okamoto M, Nath Roy C, et al Detection of enterovirus 68 in serum from pediatric patients with pneumonia and their clinical outcomes. Influenza Other Respir Viruses. 2014; 8: 21–24. 10.1111/irv.12206 24209770 PMC4177794

[12] Imamura T, Suzuki A, Lupisan S, Okamoto M, Aniceto R, Egos RJ, et al Molecular evolution of enterovirus 68 detected in the Philippines. PLoS ONE. 2013; 8: e74221 10.1371/journal.pone.0074221 24073203 PMC3779236

[13] Linsuwanon P, Puenpa J, Suwannakarn K, Auksornkitti V, Vichiwattana P, Korkong S, et al Molecular epidemiology and evolution of human enterovirus serotype 68 in Thailand, 2006–2011. PLoS ONE. 2012; 7: e35190 10.1371/journal.pone.0035190 22586446 PMC3346751

[14] Piralla A, Girello A, Grignani M, Gozalo-Margüello M, Marchi A, Marseglia G, et al Phylogenetic characterization of enterovirus 68 strains in patients with respiratory syndromes in Italy. J Med Virol. 2014; 86: 1590–1593. 10.1002/jmv.23821 24155220 PMC7166609

[15] Jacobson LM, Redd JT, Schneider E, Lu X, Chern S-WW, Oberste M, et al Outbreak of lower respiratory tract illness associated with human enterovirus 68 among American Indian children. Pediatr Infect Dis J. 2012 31: 309–312. 10.1097/INF.0b013e3182443eaf 22315004

[16] Centers for Disease Control and Prevention (CDC). Clusters of acute respiratory illness associated with human enterovirus 68—Asia, Europe, and United States, 2008–2010. MMWR Morb Mortal Wkly Rep. 2011; 60: 1301–1304. 21956405

[17] Jaramillo-Gutierrez G, Benschop KSM, Claas ECJ, de Jong AS, van Loon AM, Pas SD, et al September through October 2010 multi-centre study in the Netherlands examining laboratory ability to detect enterovirus 68, an emerging respiratory pathogen. J Virol Methods. 2013; 190: 53–62. 10.1016/j.jviromet.2013.02.010 23458694

[18] de Almeida MB, Zerbinati RM, Tateno AF, Oliveira CM, Romão RM, Rodrigues JC, et al Rhinovirus C and respiratory exacerbations in children with cystic fibrosis. Emerging Infect Dis. 2010; 16: 996–999. 10.3201/eid1606.100063 20507756 PMC3086221

[19] Pelletier J, Sonenberg N. Internal initiation of translation of eukaryotic mRNA directed by a sequence derived from poliovirus RNA. Internal initiation of translation of eukaryotic mRNA directed by a sequence derived from poliovirus RNA. Nature. 1988; 334: 320–325. 2839775 10.1038/334320a0

[20] Kaida A, Kubo H, Sekiguchi J-I, Kohdera U, Togawa M, Shiomi M, et al Enterovirus 68 in children with acute respiratory tract infections, Osaka, Japan. Emerging Infect Dis. 2011; 17: 1494–1497. 10.3201/eid1708.110028 21801632 PMC3381549

